# BMP4 Is a Peripherally-Derived Factor for Motor Neurons and Attenuates Glutamate-Induced Excitotoxicity *In Vitro*

**DOI:** 10.1371/journal.pone.0058441

**Published:** 2013-03-05

**Authors:** Hui-Ju Chou, Dar-Ming Lai, Cheng-Wen Huang, Ian S. McLennan, Horng-Dar Wang, Pei-Yu Wang

**Affiliations:** 1 Graduate Institute of Brain and Mind Sciences, College of Medicine, National Taiwan University, Taipei, Taiwan, R.O.C.; 2 Institute of Neuroscience and Research Center for Mind, Brain and Learning, National Chengchi University, Taipei, Taiwan, R.O.C.; 3 Division of Neurosurgery, Department of Surgery, National Taiwan University Hospital, Taipei, Taiwan, R.O.C.; 4 Institute of Biotechnology, National Tsing Hua University, HsinChu, Taiwan, R.O.C.; 5 Institute of Systems Neuroscience, National Tsing Hua University, HsinChu, Taiwan, R.O.C.; 6 Department of Life Science, National Tsing Hua University, HsinChu, Taiwan, R.O.C.; 7 Department of Anatomy, University of Otago, Dunedin, New Zealand; Academia Sinica, Taiwan

## Abstract

Bone morphogenetic proteins (BMPs), members of the transforming growth factor-beta (TGF-β) superfamily, have been shown to play important roles in the nervous system, including neuronal survival and synaptogenesis. However, the physiological functions of BMP signaling in the mammalian neuromuscular system are not well understood. In this study, we found that proteins of the type II bone morphogenetic receptors (BMPRII) were detected at the neuromuscular junction (NMJ), and one of its ligands, BMP4, was expressed by Schwann cells and skeletal muscle fibers. In double-ligated nerves, BMP4 proteins accumulated at the proximal and distal portions of the axons, suggesting that Schwann cell- and muscle fiber-derived BMP4 proteins were anterogradely and retrogradely transported by motor neurons. Furthermore, BMP4 mRNA was down-regulated in nerves but up-regulated in skeletal muscles following nerve ligation. The motor neuron-muscle interactions were also demonstrated using differentiated C2C12 muscle cells and NG108-15 neurons *in vitro*. BMP4 mRNA and immunoreactivity were significantly up-regulated in differentiated C2C12 muscle cells when the motor neuron-derived factor, agrin, was present in the culture. Peripherally-derived BMP4, on the other hand, promotes embryonic motor neuron survival and protects NG108-15 neurons from glutamate-induced excitotoxicity. Together, these data suggest that BMP4 is a peripherally-derived factor that may regulate the survival of motor neurons.

## Introduction

In the neuromuscular system, a dynamic interaction occurs among motor neurons, Schwann cells and muscle fibers. Motor neuron-derived agrin, for instance, can induce the formation of the neuromuscular junction (NMJ) [1], [2], while signals from skeletal muscle fibers and Schwann cells are able to regulate the survival of motor neurons [3], [4]. The large variety of neurotrophic factors that can support motor neuron survival in culture and in animal models of nerve injury indicates that developing and postnatal motor neurons depend upon cooperation of these molecules [5]–[8]. Recent studies show that genetic deletion of a single, or even multiple, growth factors, only lead to a partial loss of motor neurons [9]–[11]. This implies that motor neurons may be affected by numerous muscle fiber- and Schwann cell-derived survival factors. Equally, this may also indicate that there are distinct subpopulations of motor neurons that rely on different survival factors [10].

Bone morphogenetic proteins (BMPs), the largest subgroup within the TGF-β superfamily, were originally identified by their ability to induce bone differentiation [12]. To date, more than 20 BMPs have been identified, having diverse biological functions, such as cell proliferation, differentiation, morphogenesis and apoptosis [13], [14]. Like other TGF-β superfamily members, BMPs signal through a complex involving both a type I and a type II receptor. In general, the type II receptors control the ligand-binding specificity, while the type I receptors determine which downstream signaling pathway is activated. There are five type II receptors and seven type I receptors [15]. The type II receptors associate with specific subfamilies of the superfamily and are referred to as the TGF-β, BMP, Mullerian inhibiting substance and activin A/B type II receptors. The seven type I receptors are ALK1 to ALK7.

It has been shown that BMP signaling is required for normal development of the *Drosophila* neuromuscular system. Mutations of *Glass bottom boat* (BMP homolog) and its receptors (*Wishful Thinking* for the type II receptor and *Thickveins* for the type I receptor) lead to profound defects at the *Drosophila* NMJ, such as decreased neurotransmitter release, reduced synaptic size and aberrant presynaptic ultrastructure [16]–[18]. However, the function of BMPs in the mammalian neuromuscular system remains unclear. In this study, we report BMP4 as a peripherally-derived factor that may regulate the survival of motor neurons.

## Results

### The type II BMP receptor is associated with the NMJ

**Figure 1:**
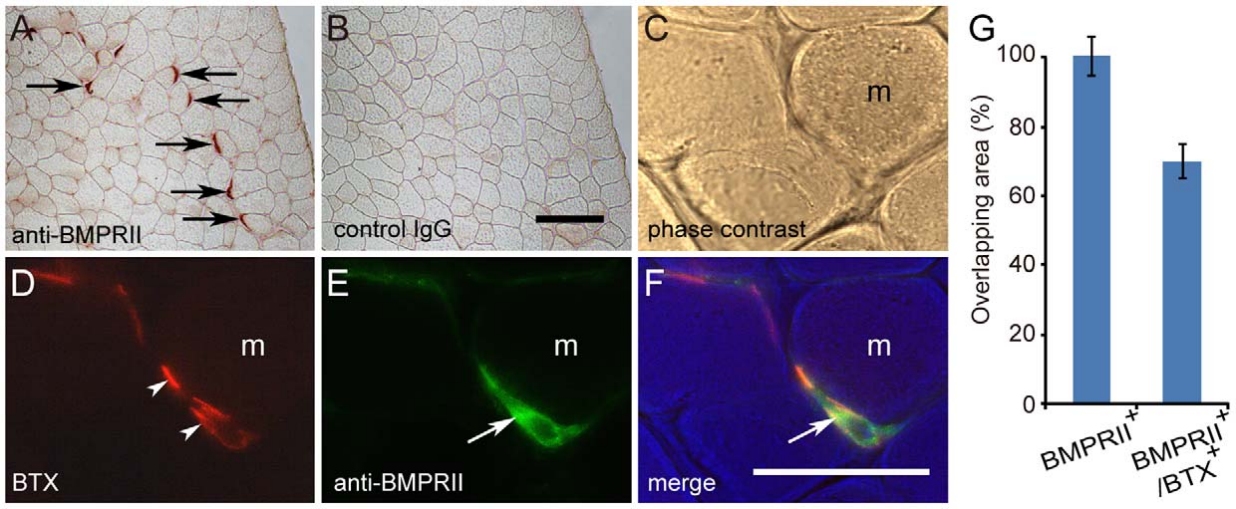
BMPRII proteins are detected at NMJs. (A and B) Adjacent sections of EDL muscle were stained with an anti-BMPRII antibody (A) and a control IgG (B) antibody. The sections were visualized using a color reaction product (AEC). (C–F) A single section of EDL muscle was stained with an anti-BMPRII antibody (E; green) and BTX (D; red) to label NMJs. Bright field image (C), BTX in red (D) and BMPRII in green (E) channels were merged using Adobe Photoshop software (F). The arrows identify BMPRII immunoreactivity. Arrowheads point to the NMJs labeled by BTX. “m” indicates muscle fibers. Scale bars  = 100 μm (A, B) and 50 μm (C–F). (G) The overlapping area for BMPRII immunoreactivity and BTX signal was quantified using Image J software. Values are mean ± SEM (n = 21).

We have previously shown that BMPRII mRNA and protein were detected in the cell bodies of lumbar spinal motor neurons [19]. We further examined the expression of BMPRII in other parts of the neuromuscular system. In the EDL muscle and soleus muscle (not illustrated), strong BMPRII immunoreactivity was associated with many muscle fibers (Fig. 1A, E and F). To examine whether the BMPRII immunoreactivity was associated with NMJs, sections were also co-stained with BTX to label NMJs. Approximately 71.7% of the BMPRII immunoreactivity was found to overlap with BTX-labeled NMJs (Fig. 1C–G). BMPRII immunoreactivity, however, was not detected in sciatic nerves (data not shown).

### BMP4 mediates motor neuron and muscle interactions

**Figure 2:**
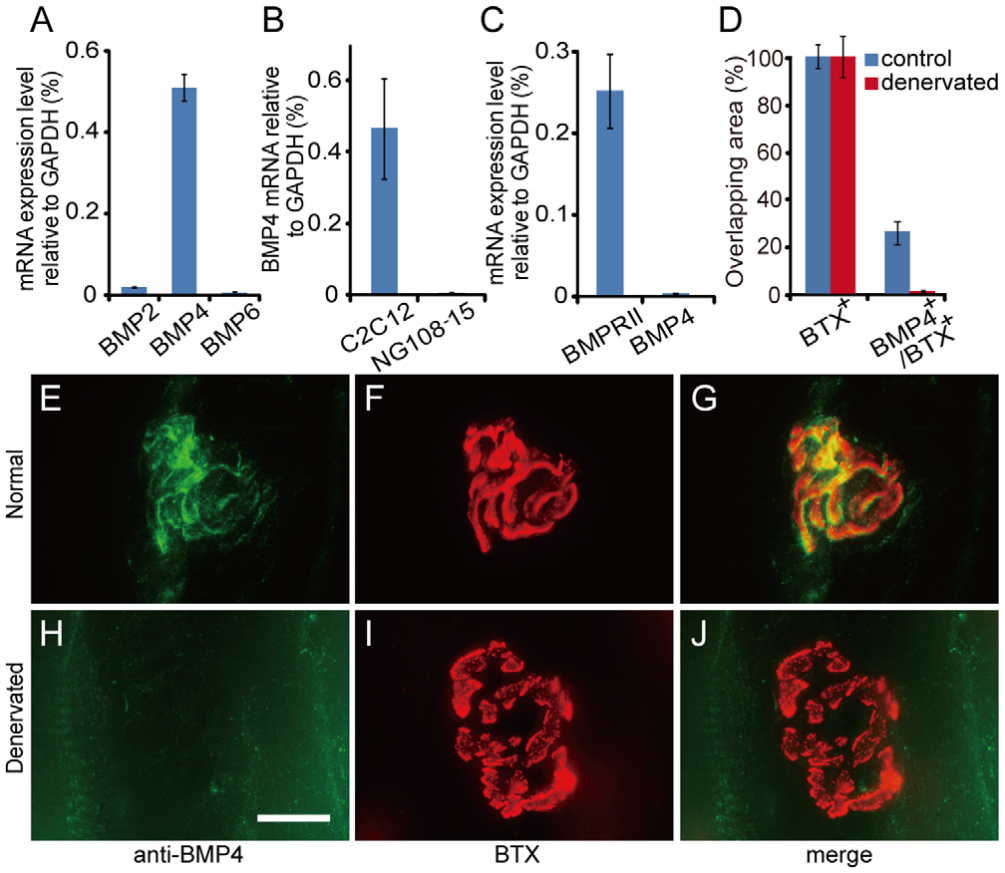
BMP4 is expressed by muscles. (A) Real-time PCR measurements of BMP2, BMP4 and BMP6 mRNA expression in differentiated C2C12 muscle cells. (B) Real-time PCR measurements of BMP4 mRNA expression in differentiated C2C12 muscle cells and NG108-15 neurons. (C) Real-time PCR measurements of BMPRII and BMP4 mRNA expression in laser capture-isolated lumbar spinal motor neurons. BMPRII mRNA measurement is shown as a positive control here. Data are presented using GAPDH expression level as 100%. Values are mean ± SEM (n = 6 for A, n = 11 for B and n = 8 for C). (E–J) Localization of BMP4 protein (green) in normal (E–G) and denernvated (H–J) soleus muscles was examined by immunohistochemistry. NMJs were labeled by BTX (red). Grouped panels E, F, G and H, I, J, show single sections of normal or denervated soleus muscle, respectively, stained for BMP4 in green (E and H) and for BTX in red (F and I) channels. Green and red channels were merged using Adobe Photoshop software (G and J). Scale bar  = 15 μm (E–J). (D) The overlapping area for BMP4 immunoreactivity and BTX signal in normal and denervated muscles was quantified using Image J software. Values are mean ± SEM (n = 12).

The association of BMPRII proteins with the NMJs indicated that motor neurons may receive a muscle-derived BMP. We further investigated whether BMP2, BMP4 or BMP6 is a possible candidate for mediating motor neuron and muscle interactions. Differentiated NG108-5 neurons and C2C12 muscle cells were used here because they are capable of forming functional neuromuscular synapses when co-cultured [20]. We found that BMP4 mRNA had a much higher expression level in differentiated C2C12 muscle cells compared to BMP2 and BMP6 (Fig. 2A), and was barely detectable in NG108-15 neuron cells (Fig. 2B). A lower expression level of BMP4 mRNA was also confirmed in lumbar spinal motor neurons. In the laser capture-isolated motor neuron samples, only a very low level of the BMP4 mRNA could be detected, while BMPRII mRNA as a positive control, showed a much higher expression level (Fig. 2C). These results led us to further investigate the function of BMP4 in the neuromuscular system.

Localization of the BMP4 protein in the soleus muscle was examined using immunohistochemistry. BMP4-immunoreactivity was detected in muscle fibers, where it was expressed mainly in the vicinity of BTX-labeled NMJs, with only 26.3% of the BTX signal co-localizing with BMP4-immunoreactivity (Fig. 2D–G). However, in the denervated soleus muscle (Fig. 2D, H–J), disappearance of BMP4 immunoreactivity following denervation indicates that expression of BMP4 in muscle cells may be regulated by a motor neuron-derived factor.

**Figure 3:**
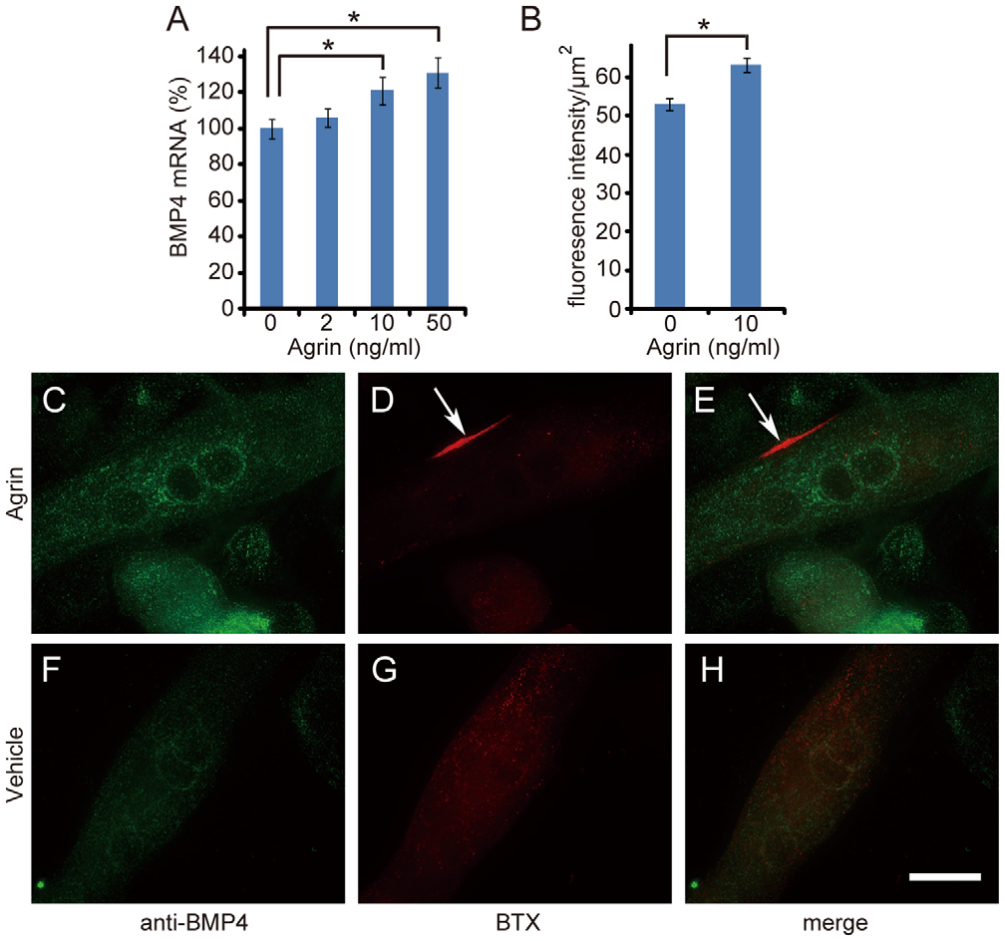
BMP4 mRNA and protein expression are up-regulated by agrin. Differentiated C2C12 muscle cells were treated with agrin, or vehicle for 16 hours. BMP4 mRNA expression level and protein localization were examined by real-time PCR (A) and immunocytochemistry (C–H). Green fluorescence intensity of the BMP4 immunoreactivity (C and F) was quantified using Image J software (B). NMJs (red, arrows) were labeled with BTX (D and G). Green and red channels were merged using Adobe Photoshop software (E and H). Values are mean ± SEM (n = 6 samples for A, and n = 70 myotubes for B; *, *P*<0.05, compared to controls using Student's *t* test). Scale bar  = 15 μm (C–H).

Agrin is a well known clustering agent for AchR. It is synthesized by motor neurons, anterogradely transported, and released at the nerve terminals [2], [21]. We examined whether agrin can also affect BMP4 expression (Fig. 3A and B) or its localization (Fig. 3C–H) at the NMJ. Addition of agrin to the medium of differentiated C2C12 muscle cells caused AChR to aggregate on the surface of the myotube (Fig. 3D and E). Agrin caused a dose-dependent increase in mRNA expression (Fig. 3A) and in immunoreactivity for BMP4 (Fig. 3B, C and F) in differentiated C2C12 muscle cells, observations that were not seen when agrin was absent from the culture medium (Fig. 3F–H). This increase appeared to be BMP4-specific, since BMP2 and BMP6 mRNA expression in differentiated C2C12 muscle cells was not affected by agrin (data not shown). Additionally, localization of BMP4 protein in differentiated C2C12 muscle cells was not affected by agrin, suggesting that targeting of BMP4 to the NMJ may be regulated by other factors that require further investigation (Fig. 3C–H).

### BMP4 is produced by Schwann cells and transported in the motor neurons

**Figure 4:**
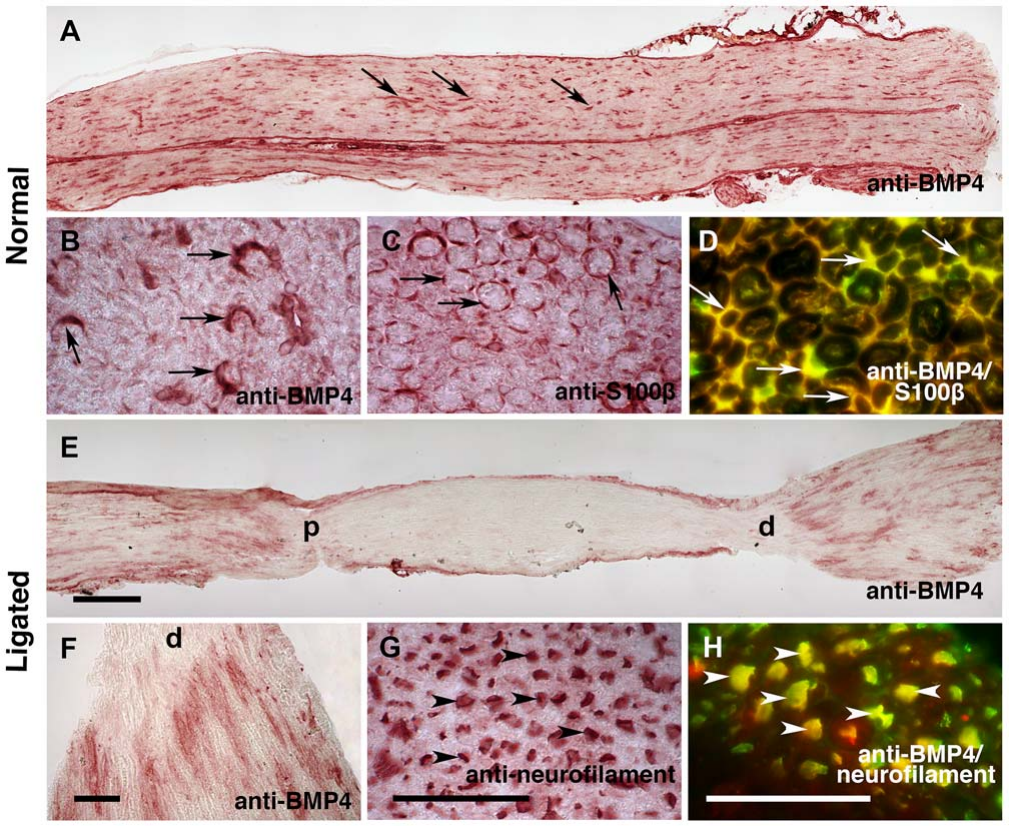
BMP4 is produced by Schwann cells and transported by motor neurons. (A–D) Normal sciatic nerves were cut into longitudinal (A) or cross (B–D) sections. Sections were stained with an anti-BMP4 antibody (A and B), or an anti-S100βantibody that labels myelin sheaths of Schwann cells (C), and visualized using a color reaction product (AEC). (D) A single section was double-stained with anti-BMP4 (red) and anti-S100β (green) antibodies to visualize co-localization of BMP4 immunoreactivity and Schwann cell staining. Red and green channels were merged using Adobe Photoshop software. (E–H) Double-ligated sciatic nerves were cut into longitudinal (E and F) or cross (G and H) sections. The sections were stained with an anti-BMP4 antibody (E and F), or an anti-neurofilament antibody to mark axons (G), and visualized using a color reaction product (AEC). The proximal and distal ligations are marked in (E) by “p” and “d”, respectively. (F) Higher magnification of the proximal region illustrated in (E). (H) A single section was double-stained with anti-BMP4 (red) and anti-neurofilament (green) antibodies to show colocalization of BMP4 immunoreactivity and axons. Red and green channels were merged using Adobe Photoshop software. The arrows and arrowheads point to Schwann cells and axons, respectively. Magnifications in A and E; B, C and G; D and H are the same. Scale bars  = 200 µm (A and E) and 50 µm (B–D, F–H).

Localization of BMP4 protein in sciatic and hypoglossal nerves was examined by immunohistochemistry. Intense BMP4-immunoreactivity was detected along longitudinal sections of the sciatic nerve (Fig. 4A) and the hypoglossal nerve (not illustrated). Cross sections of the nerves further confirmed that BMP4 immunoreactivity was associated with the semi-concentric-like structure of Schwann cell myelin sheaths (Fig. 4B–D). If muscle fibers and Schwann cells use BMP4 to regulate the function of motor neurons, BMP4 should be axonally transported, like other neurotrophic factors [19], [22], [23]. The nerves were therefore double ligated in order to determine whether BMP4 was transported in the axon. We found BMP4-immunoreactivity associated with both the proximal and distal portions of ligation sites in these nerves (Fig. 4E and F), indicating that BMP4 was being anterogradely and retrogradely transported. The immunoreactivity at both ligation sites appeared to be in axons. This was further verified by cutting cross sections of the nerves, which allows for clear delineation between axonal and Schwann cell proteins [19], [22], [23]. In cross sections of the double ligated nerve, the motor axons can be unambiguously identified (Fig. 4G), and some of the motor axons contained intense BMP4-immunoreactivity that overlapped with anti-neurofilament labeled axons (Fig. 4H).

### Axon damage affects peripheral BMP4 expression

**Figure 5:**
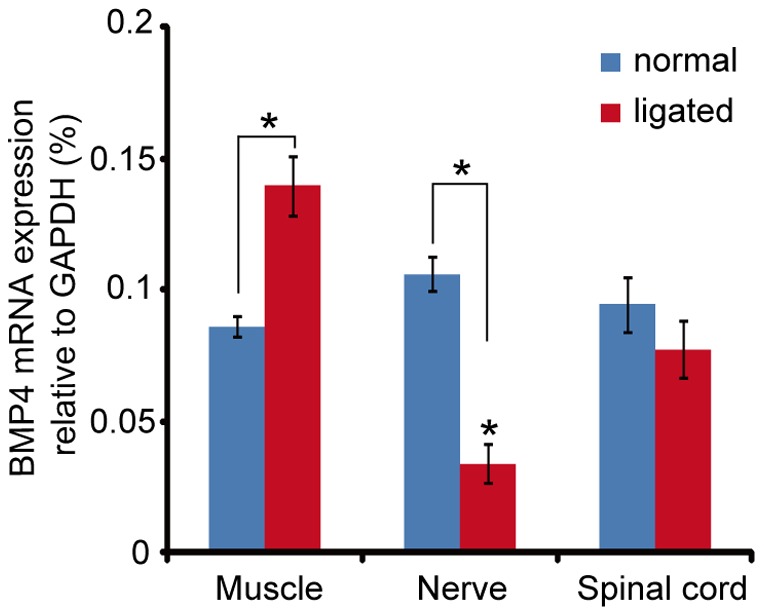
Expression of BMP4 mRNA in the neuromuscular system following nerve ligation. Total RNA was isolated from sciatic nerve (n = 4), soleus muscle (n = 6) and lumbar spinal cord (n = 6) of control and nerve-ligated mice. Expression levels of BMP4 mRNA were measured by real-time PCR. Data are presented using GAPDH expression level as 100%. Values are mean ± SEM (*, *P*<0.05, compared to normal mice using Student's *t* test).

Many peripherally-derived factors are known to change their expression pattern after nerve injury, and these responses are thought to be involved in the regeneration process of nerves [22], [24]–[26]. After 18 to 20 hours of sciatic nerve ligation, BMP4 mRNA was significantly up-regulated in the soleus muscle (Fig. 5). However, BMP4 mRNA was down-regulated in ligated sciatic nerves themselves, and no difference was observed in the lumbar region of the spinal cord (Fig. 5).

### BMP4 may act as a survival factor for motor neurons

**Figure 6:**
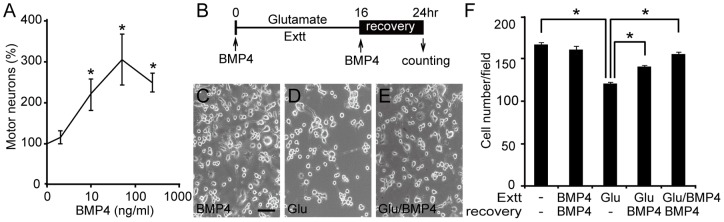
BMP4 promotes embryonic motor neuron survival and protects NG108-15 neurons against glutamate (Glu)-induced excitotoxicity (Extt). (A) A dose-response curve for the survival effect of BMP4 on embryonic motor neurons. The numbers of surviving motor neurons were determined by counting islet-1 positive neurons under a microscope. Data are presented using cell counts in the vehicle group as 100%. (B–F) Differentiated NG108-15 cells were treated with glutamate and/or BMP4 for 16 hours, followed by an 8-hour recovery period in conditioned medium with or without BMP4. Three representative photos show the morphology of differentiated NG108-15 neurons in control (C), glutamate-treated (D) and glutamate plus BMP4-treated (E) conditions. Scale bars  = 100 µm. The numbers of surviving neurons were counted under a microscope by trypan blue exclusion (F). Cell numbers from at least 10 fields (A) and 60 fields (F) for each group were counted. Values are mean ± SEM (*, *P*<0.05, by Student's *t* test).

BMP4 was found to be expressed by peripheral cells and axonally transported by motor neurons. These properties suggested that BMP4 may act like a classic peripherally-derived survival factor for motor neurons. We therefore examined the survival effect of BMP4 on isolated embryonic motor neurons and differentiated NG108-15 neurons *in vitro*. In the embryonic motor neuron cultures, addition of BMP4 to the medium induced a dose-dependent increase in neuronal survival, with the maximum effect found at a concentration of 10 to 50 ng/ml (Fig. 6A). We further tested the protective effect of BMP4 on glutamate-induced excitotoxicity. Differentiated NG108-15 neurons were exposed to glutamate (50 μM) for 16 hours, followed by an 8-hour recovery period (conditioned medium), and the effects of BMP4 were examined by counting the number of surviving neurons (Fig. 6B). Addition of BMP4 to the conditioned medium during the recovery period significantly protected NG108-15 neuronal cells from glutamate-induced cell death (Fig. 6C–F). Greater protection could be achieved by addition of BMP4 to the cultures during both glutamate exposure and recovery periods (Fig. 6F). The presence of BMP4 alone in the cultures did not affect the survival of neurons (Fig. 6F).

## Discussion

### BMP4 as a physiological regulator for motor neurons

In this study we have demonstrated that the BMP family members are important regulators for motor neurons. The identification of BMPRII and BMP4 in the neuromuscular system suggests that BMP4 may mediate motor neuron-peripheral interactions. This is in agreement with previous studies using fruit flies as a model for studying the neuromuscular system. Strong connections among BMP signaling, synaptic growth and synaptic stabilization at *Drosophila* NMJ have already been established [16]–[18].

Our data suggest that BMP4 is a peripherally-derived factor for motor neurons. Its mRNA was present in muscles and nerves (Fig. 2, 3 and 5), and BMP4 immunoreactivity was also detected in Schwann cells and in the vicinity of NMJs (Fig. 2 and 4). Most importantly, ligation of sciatic and hypoglossal nerves led to the accumulation of BMP4 proteins at both proximal and distal tie (Fig. 4). This implies that there is a continuous flow of BMP4 up and down the motor axons. The characteristics of peripheral expression and axonal transport are shared by BMP4 and other peripherally-derived neurotrophic factors such as BMP6 [19], glial cell line-derived neurotrophic factor (GDNF) [23] and TGF-β2 [22].

BMP4 and BMP6 both signal through BMPRII and other BMP type I receptors [15]. This may raise the possibility of functional redundancy of BMP4 and BMP6 with respect to motor neurons. In fact, we have shown that both BMP4 and BMP6 [19] were produced by Schwann cells and were able to support motor neuron survival *in vitro*. BMP4 and BMP6, nevertheless, may also regulate distinct functions in the neuromuscular system, as only BMP4 is expressed in adult muscle cells, while BMP6 is mainly produced in developing myotubes. BMP4 and TGF-β2 are anterogradely and retrogradely transported by motor neurons [22], while BMP6 is largely transported towards the cell bodies of motor neurons [19], and GDNF is mainly transported towards the nerve terminal [23]. It is not clear why so many peripherally-derived factors are used to communicate with motor neurons. One reasonable explanation is that the peripheral cells may use different factors in different contexts to regulate different aspects of motor neuron function.

### Regulation of muscle-derived BMP4

Muscle-derived signals are essential for both developing and mature motor neurons. Although the target dependence only exists until early postnatal stage (P7 to P10) for the survival of developing motor neurons [27], mature motor neurons continue to receive some neurotrophic signals from adult muscle fibers. For instance, adult motor neurons receive muscle derived neurotrophin-4 (NT-4) for nerve growth and remodeling, although the expression of NT-4 in muscle fibers requires signals from motor neurons [28]. Equally, the disappearance of BMP4 immunoreactivity in muscle fibers following nerve transaction may suggest that BMP4 expression is regulated by motor neurons in a fashion similar to that of NT-4 (Fig. 2). In addition to agrin, we speculate that other unknown factors may be involved in this process. The neuronal regulation of BMP4 may reflect its function in the regulation of neuron survival, axon growth, and maintenance of synaptic connections as reported for NT-4 previously [28], [29]. Although it is not clear how agrin regulates BMP4 expression in muscle cells, the activation of Lrp4/Musk receptor complex by agrin has been shown to stimulate JNK pathways which may further induce BMP4 expression [30], [31].

The up-regulation of BMP4 in skeletal muscles after nerve ligation could reflect a similar physiological function for BMP4 to that discussed above. Indeed, it has been shown that enhanced BMP4 signaling at the dorsal root ganglion is able to induce axon regeneration in an animal model of spinal injury [32]. The actions of BMP4 are, nevertheless, context-dependent, as manipulation of the BMP pathway locally at injury sites affects astrogliosis [33], and may inhibit axon regeneration [34].

### Regulation of Schwann cell-derived BMP4

Extensive cell death occurs in mature motor neurons only when they are deprived of both muscles and Schwann cells by ventral root avulsion [35]. This indicates that Schwann cells are at least as important as the target muscles for motor neuron survival. Our studies imply that BMP4 can also derive from Schwann cells. In comparison to injured nerves, relatively higher levels of BMP4 mRNA and protein are present in healthy nerves (Fig. 4 and 5), suggesting that Schwann cells could use BMP4 to communicate with motor neurons on a day-to-day basis. Muscle-derived BMP4, in contrast, is up-regulated following nerve damage, pointing to a possible role as a specialized injury factor that may be responsible for axonal regeneration (discussed above).

The potential of Schwann cell-derived BMP4 as a day-to-day regulator is analogous to the observed in ciliary neurotrophic factor (CNTF). In adult rats, CNTF is normally expressed at high levels in Schwann cells, but is reduced dramatically after nerve damage [24]. Mice and humans with mutations of CNTF display a progressive loss of motor neurons, and may have a higher risk of developing more severe motor neuron disease [36], [37]. These studies together support the hypothesis that CNTF and BMP4 act as critical modifiers for motor neuron disease. In fact, recent studies lend additional support to our view, demonstrating that dysregulation of BMP signaling could be one of the causes of axonopathy in human hereditary spastic paraplegias [38].

### Is BMP4 a motor neuron survival factor?

Classical survival factors for motor neurons are derived from their peripherals. The characteristics of BMP4 fit most criteria for being a physiological survival factor for motor neurons, although our *in vitro* experiments can only partial answer this question (Fig. 6). Motor neurons are particularly vulnerable to glutamate-induced cell death, and the inhibition of glutamate release with riluzole can prolong survival of patients with motor neuron diseases [39]. Evidence for excitotoxicity in motor neuron diseases also includes elevated glutamate levels in the cerebrospinal fluid of a subset of patients [40]. It is known that glutamate excitotoxicity induces an acute morphological change and is followed by a massive cell death at the later stages [41]. The protective effects of BMP4 seen during both glutamate exposure and recovery periods in this study may provide for a better intervention for treating motor neuron and other neurodegenerative diseases in the future.

In summary, our data support a model that peripheral cells may use BMP4 to communicate with motor neurons. The extent to which peripherally-derived BMP4 affects motor neurons during normal and pathological physiology is not fully understood. Here, we provide some evidence showing that BMP4 may be involved in the survival regulation of motor neurons. Future studies will utilize animal models where BMP4 and BMPRII can be specifically deleted in each cell type. Such experiments will help to further delineate the actions of BMP4 in the neuromuscular system.

## Materials and Methods

### Animals

The National Chengchi University's Animal Ethics Committee approved all experiments. C57Bl6 mice were bred and maintained in the Modular Animal Caging System® (Alternative Design) and their food sterilized by gamma irradiation. The room had a 12 h light/12 h dark phase, with the dark phase beginning at 8 pm.

### Isolation of motor neuron RNA

Motor neuron mRNA was isolated using laser capture microdissection as previously described [42]. Briefly, lumbar spinal cords were sectioned in a cryostat, stained with cresyl violet, and their motor neurons harvested using the PixCell 2 LCM System and CapSure HS LCM Caps (Arcturus Engineering). Typically, 4–500 motor neurons were collected from each mouse. Purity of the mRNA was confirmed by measuring the abundance of a glial marker, glial fibrillary acidic protein.

### RNA preparation, cDNA synthesis, and real-time PCR

Total RNA was prepared using the RNeasy Mini Kit (Qiagen). The RNA was treated with DNase and converted to cDNA using oligo-d(T)_15_ (Invitrogen) and SuperScript III reverse transcriptase (Invitrogen) as described previously [43]. Real-time PCR reactions were performed using a 7300 Real-Time PCR System (Applied Biosystems), SYBR Green Master Mix (Applied Biosystems) and gene-specific primers were CTTCATTGACCTCAACTA and TTCACACCCATCACAAAC for GAPDH; CAGCTGCAAGAGACACCCTTTGTAT and ATGCCTTAGGGATTTTGGAATTCA for BMP2; TCGCCATTCACTATACGTGGACTT and CACAACAGGCCTTAGGGATACTAGA for BMP4; GCCATCTCGGTTCTTTACTTCGAT and GTGGTTTAAGGCAGATGTTGTTGTT for BMP6; AGGATCAGGTGAAAAGATCAAGAGA and GCAAGGTACACAGCAGTGCTAGATT for BMPRII; GTGCTCCTGCTTCGAGTCCTTAA and AACCGCATCACCATTCCTGTACA for GFAP. A two-step PCR reaction was carried out with denaturation at 95°C for 15 seconds, annealing and extension combined at 60°C for one minute in a total of 40 cycles. The mRNA expression level of each target gene compared to GAPDH was quantified by subtraction: Ct_target_ – Ct_GAPDH_  =  ΔCt. A difference of one PCR cycle equates to a 2-fold change in mRNA expression level. Data were calculated using GAPDH expression level as 100%. The uniqueness of amplicons was analyzed using dissociation curves.

### Operations

Adult mice were anaesthetized with isoflurane gas (2–3% by volume, 0.4 L/min). The left hypoglossal or sciatic nerve was double ligated as previously described [22]. A midline incision was made in the ventral neck and the tendon of the digastric muscle sectioned to expose the hypoglossal nerve. The nerve was then ligated in two places, 1–2 mm apart, using fine surgical thread. The sciatic nerve was exposed by separating the anterior border of the biceps femoris from other structures and ligated as described for the hypoglossal nerve. Eighteen to 20 hours after ligation, the animals were killed in a CO_2_ chamber and their nerves dissected. In some experiments, the extensor digitorum longus (EDL), soleus, and tibialis anterior muscles were denervated by sectioning the sciatic nerve in one of the hind limbs, as previously described [44]. The mice were killed by cervical dislocation 7 days after the operation. The muscles were removed and either processed for RNA analysis or snap frozen in melting isopentane for immunohistochemical analysis.

### Immunostaining

The whole-mount tissues, cryosections or culture cells were stained by immunohistochemistry and immunocytochemistry, as previously described [19], [23]. Antibodies included anti-BMP4 (Santa Cruz Biotechnology), anti-BMPRII (R&D System), anti-synaptophysin (Abcam), anti-neurofilament 200 (Sigma), anti-S100 beta (Abcam). Immunoreactivity was visualized using fluorescent Alexa Fluor 350, Alexa Fluor 488 and BODIPY-fl conjugated secondary antibodies (Invitrogen) or using 3-amino-9-ethylcarbamide (AEC) (Sigma) as the chromogen. NMJs were labeled with rhodamine-conjugated-α-bungarotoxin (BTX, Molecular Probes). Non-specific binding was controlled for by replacing the primary antibody with non-immune IgG (Sigma). The immunofluorescent intensity and overlapping area was quantified using Image J software.

### Cell cultures

The embryonic motor neuron cultures were performed as described previously [42]. Recombinant human BMP4 (R&D System) was added immediately after seeding. Half the volume of medium was changed after 2 days. Four days after plating, the cultures were stained with antibody to the motor neuron marker, anti-islet-1 (39.4D5, Developmental Studies Hybridoma Bank), and the immunoreactivity was developed as above, by using biotinylated-anti-mouse IgG (Jackson ImmunoResearch). The numbers of surviving motor neurons were determined by counting islet-1 positive neurons under a microscope (10× eyepiece and 20× objective lens). Mouse muscle cells (C2C12) and neuroblastoma × glioma hybrid cells (NG108-15) were obtained from Taiwan National Health Research Institute's Cell Bank. C2C12 cells were grown as myoblasts in medium containing Dulbecco's modified Eagle's medium (DMEM) supplemented with 20% fetal bovine serum (FBS), 100 U/ml penicillin G, and 100 μg/ml streptomycin. C2C12 myoblasts were induced to differentiate and fuse into myotubes by replacing the culture medium with DMEM and 2% horse serum. Undifferentiated NG108-15 cells were maintained in medium containing DMEM supplemented with 10% FBS, 100 μM hypoxanthine, 15 μM thymidine and 1 μM aminopterin. NG108-15 cells were induced to differentiate by adding 1 mM of dibutyryl cAMP (Sigma) to the culture medium. The differentiation of NG108-15 cells was monitored by scoring neurite-positive cells (neurite defined as processes >2× soma diameter). Normally, more than 90% of the cells were neurite-positive 2 days after induction with dibutyryl cAMP, as compared to less than 10% of neurite-positive cells in the undifferentiated culture. To test the neurotrophic effects of BMP4, differentiated NG108-15 cells were treated with 50 μM glutamate (in DMEM supplemented with 10 μM glycine) and/or 10 ng/ml BMP4 for 12 hours. Afterwards, cells were returned to their original culture medium (conditioned medium), with or without BMP4 for 8 hours. Cell viability was then determined under a microscope (10× eyepiece and 10× objective lens) by trypan blue exclusion.
